# Supplementary figures and images for: Amino Acid Polymorphisms in the Basic Region of Meq of Vaccine Strain CVI988 Drastically Diminish the Virulence of Marek’s Disease Virus
Source: Viruses. 2025 Jun 26;17(7):907. doi: 10.3390/v17070907 (PMC12298729; doi:10.3390/v17070907)

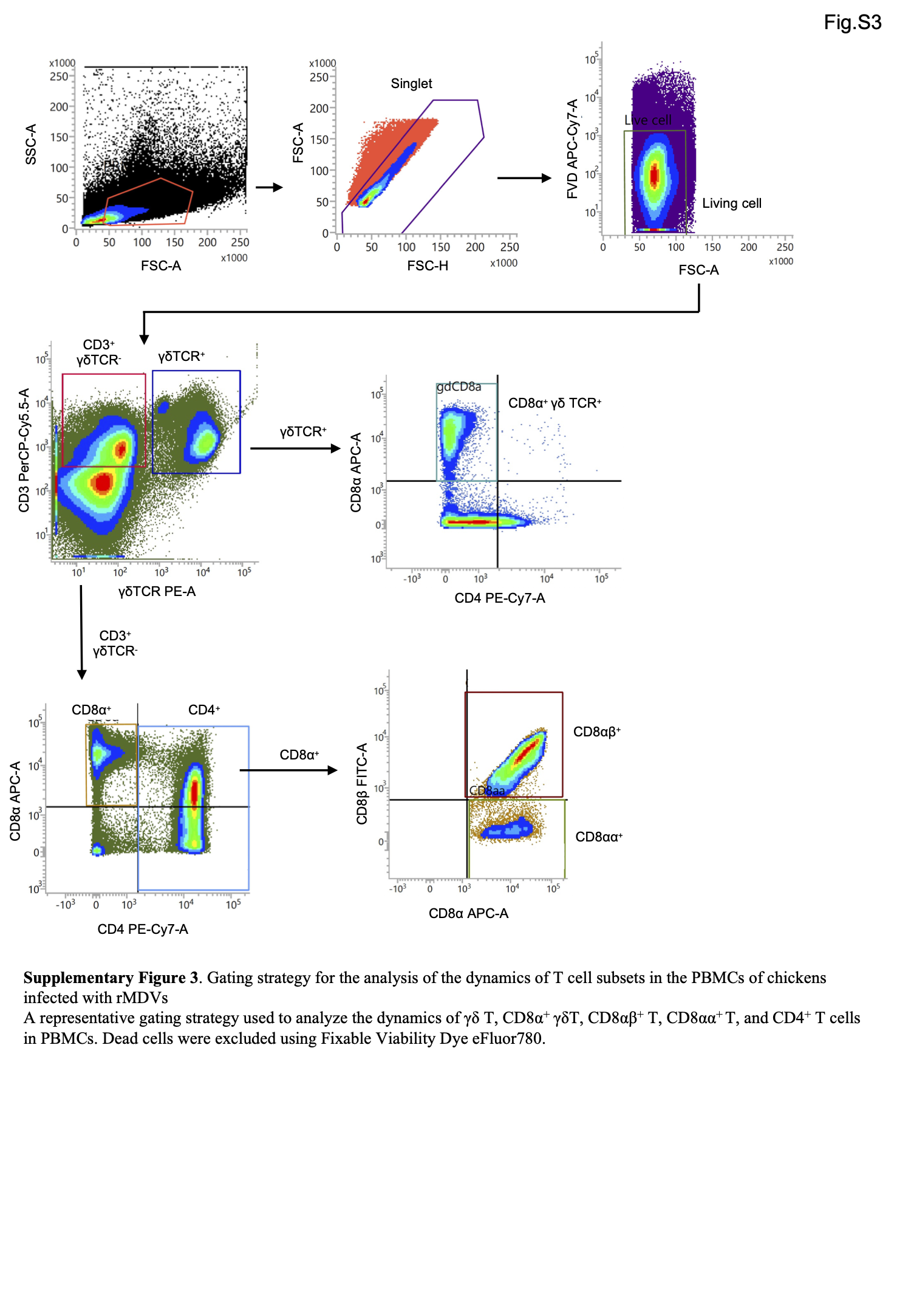

Supplement: Supplementary file 1 [file viruses-17-00907-s001.zip › Supplementary Figure 3.tiff]

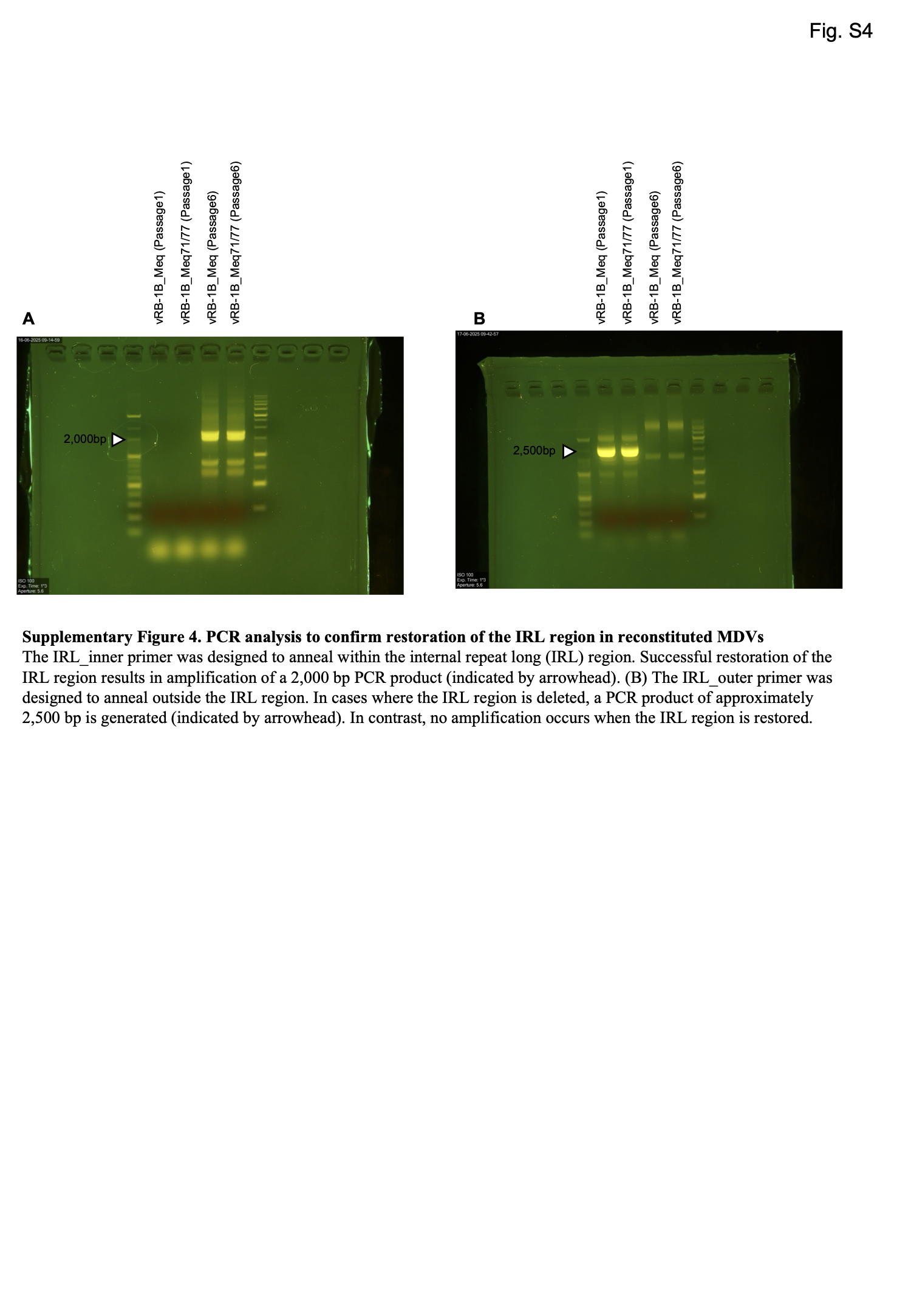

Supplement: Supplementary file 1 [file viruses-17-00907-s001.zip › Supplementary Figure 4.tiff]

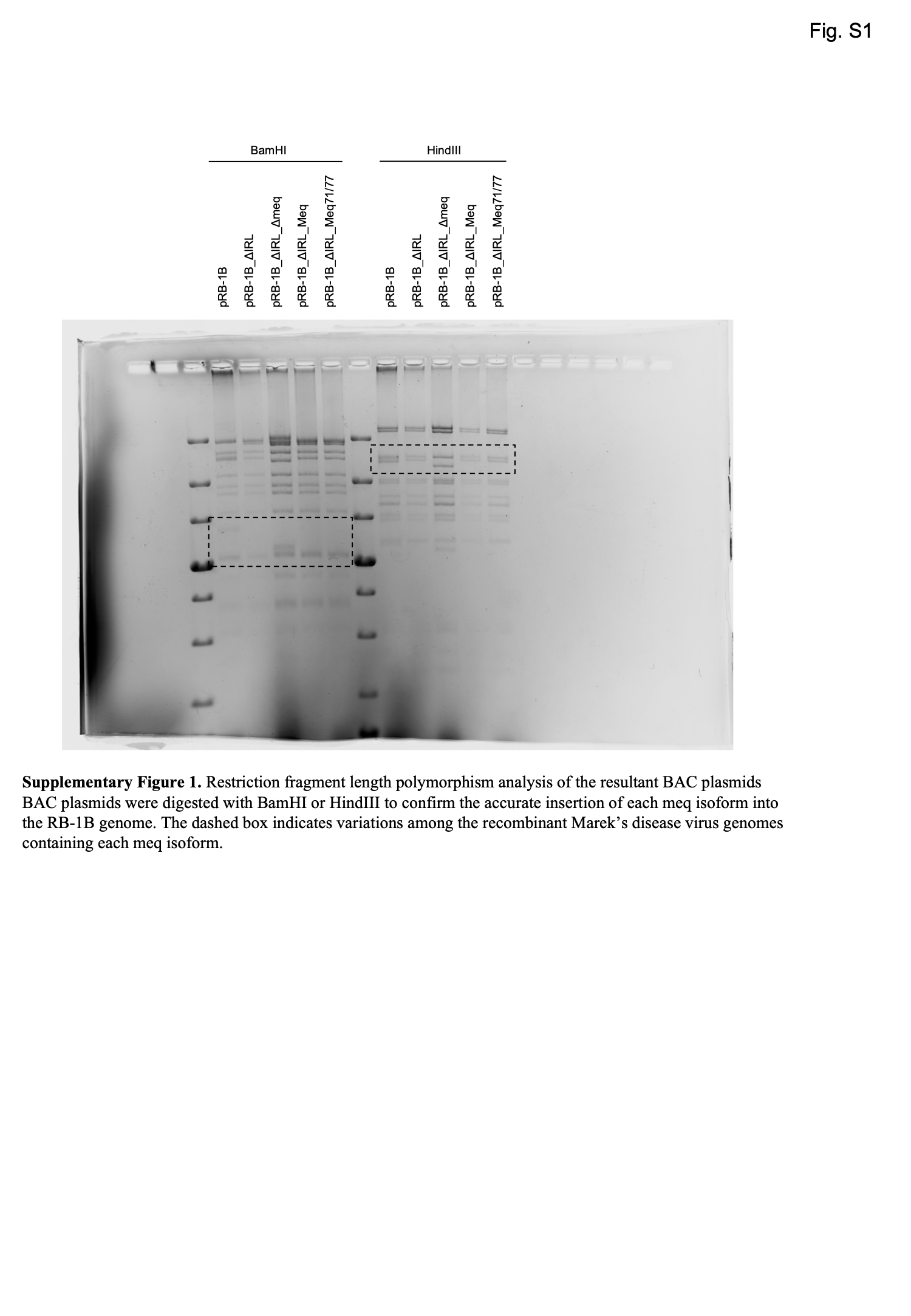

Supplement: Supplementary file 1 [file viruses-17-00907-s001.zip › Supplementary Figures 1.tiff]

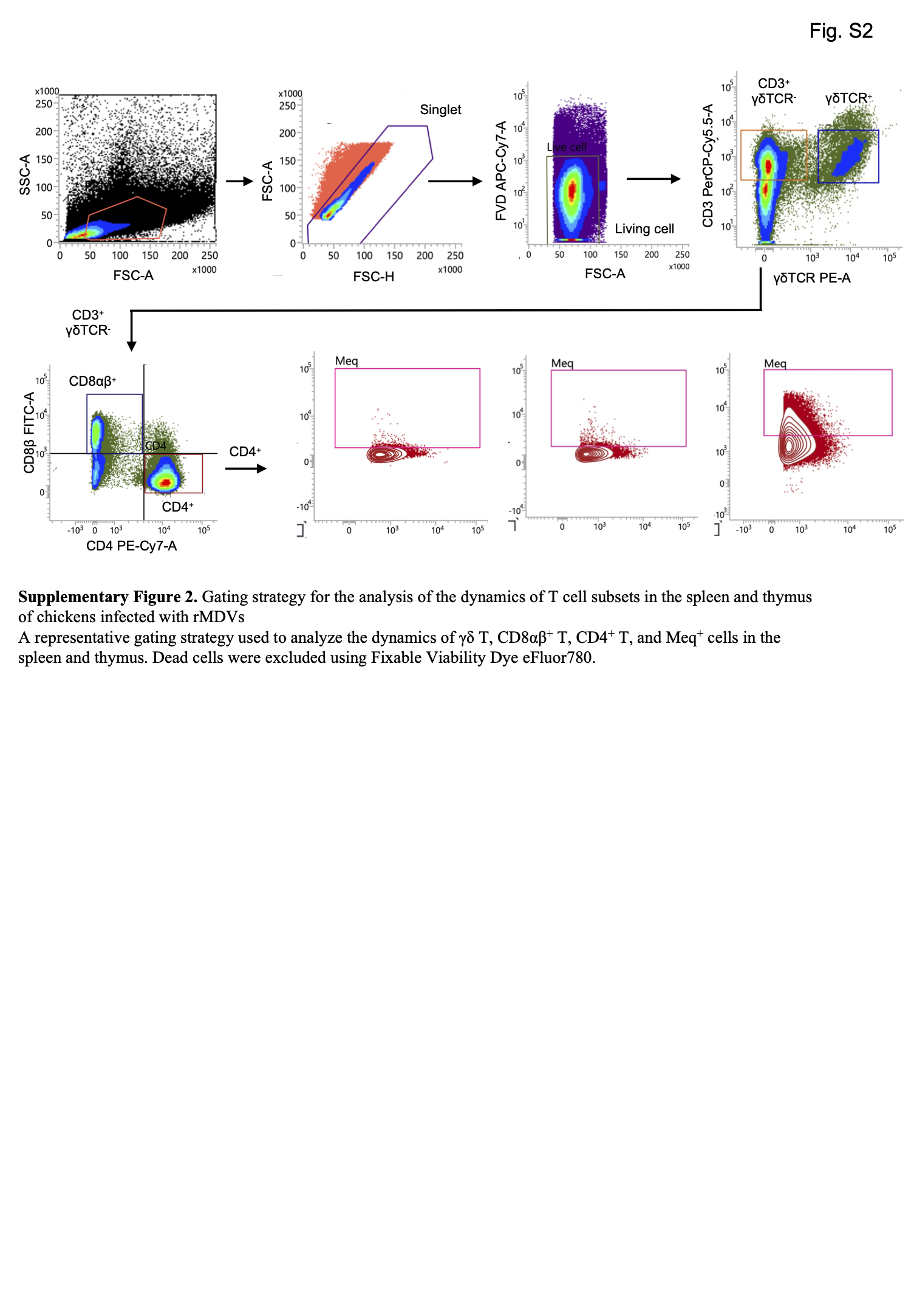

Supplement: Supplementary file 1 [file viruses-17-00907-s001.zip › Supplementary Figures 2.tiff]
